# Antiparasitic Drugs in the United States—Two Roads to High Prices

**DOI:** 10.3389/fsoc.2020.540478

**Published:** 2020-10-22

**Authors:** Arman A. Shahriar, Jonathan D. Alpern

**Affiliations:** ^1^University of Minnesota Medical School, Minneapolis, MN, United States; ^2^HealthPartners Institute, Bloomington, MN, United States; ^3^Department of Medicine, University of Minnesota, Minneapolis, MN, United States

**Keywords:** antiparasitic, drug pricing, drug costs, price hike, albendazole, miltefosine

## Abstract

High prescription drug prices contribute significantly to healthcare spending in the United States and compromise patients' access to quality medical care. A number of factors allow drug manufacturers to set much higher prices in the US than in other comparable high-income nations. Price-control depends primarily on the entry and persistence of generic products following the expiration of the market exclusivity period granted to the manufacturer of the brand name drug. Unfortunately, barriers to generic entry are common, allowing off-patent drugs like albendazole to remain relatively expensive despite having been marketed in the US for decades. By contrast, miltefosine became FDA approved more recently and has maintained a high price tag by way of a novel incentive program—the neglected tropical disease (NTD) priority review voucher (PRV) program. The voucher has a high market value and can be sold or transferred well before the drug for which it was awarded becomes available on the market. While both drugs are used to treat parasitic infections that are uncommon in the US, they differ by market and regulatory conditions—each telling an interesting pricing story.

The United States spends nearly twice as much on healthcare as other comparable high-income countries in the Organization for Economic Cooperation and Development (OECD), while performing worse by several key public health outcomes. An important driver of this difference in spending are the high prices of prescription drugs (Emanuel, 2018; Papanicolas et al., 2018). Brand-name drugs account for the majority (74%) of US prescription drug spending despite constituting only 10% of total prescriptions (Association for Accessible Medications, 2017). This is primarily due to the combination of government-granted market exclusivities (“monopoly rights”) and the limited ability of public US payers—namely Medicare and Medicaid—to limit coverage and negotiate drug prices with manufacturers. By contrast, in countries with national health insurance systems, such as Canada or the UK, a central body negotiates drug prices or rejects coverage of products if the price demanded by the manufacturer outweighs the benefit of the product (Kesselheim et al., 2016). At the level of the patient, a recent nationally representative survey found that one in four Americans have difficulty paying for prescription medications (Kaiser Family Foundation, 2019).

Once the exclusivity period for a brand-name drug ends, the entry and persistence of generic manufacturers often leads to decreased prices (Kesselheim et al., 2016). After about 2–3 years, generic drug prices generally decrease by 60–70% compared to their branded equivalents, and the degree of price decrease is strongly associated with the number of manufacturers (IMS, 2016). This “free-market” system is the primary mechanism of cost-control for drugs that no longer have market exclusivity in the US (Shrank et al., 2006; Gupya et al., 2019).

Sufficient market competition among generic manufacturers is generally a predecessor to low-cost generic drug products. However, barriers to generic entry are common and can lead to monopoly-like conditions that result in high prices. A recent analysis of over 1,000 generic drugs found a clear association between price hikes and lack of competition. On average, drugs with the lowest levels of market competition experienced a 47% price increase over the 5-year study period (Dave et al., 2017). By comparison, generic drugs with relatively high levels of competition decreased in price by an average of 32% in the same time period (Dave et al., 2017).

One reason why manufacturers may not bring a generic drug to the US market is a low financial incentive, stemming from low clinical demand for that particular drug in the US. From the standpoint of the manufacturer, the incentive to enter such a market may be outweighed by the risk: that subsequent generic entry causes a downward pressure on the price of a drug with already-low sales volumes, jeopardizing profit. Paradoxically, these low-volume generic US markets appear to have become incubators for opportunistic manufacturer behavior: a recent study of generic drug price changes between 2008 and 2016 identified a much higher prevalence of price-hikes among infrequently prescribed (low-demand) generic drugs as compared with their more-frequently prescribed counterparts (Dave et al., 2019).

The nationally-publicized case of Daraprim (pyrimethamine), the first-line drug to treat toxoplasmosis, is an example of this opportunistic behavior. In 2015, Turing Pharmaceuticals (now Vyera Pharmaceuticals) acquired the rights to Daraprim and increased the price by 5,433% in 1 day (Alpern et al., 2016). This tactic was heavily scrutinized by the public (Pollack, 2015, September 20), professional infectious disease organizations (Calderwood and Adaora, 2015), and bipartisan presidential candidates (Eunjung Cha, 2015; LoGiurato, 2015). Yet, the negative press appeared to have no impact on the price of the drug, which remains cost-prohibitive. Turing's strategy was legal and demonstrates the vulnerability of certain drug markets to exploitation by the pharmaceutical industry.

Daraprim is not unique among antiparasitic drugs approved for use in the US—a market that has become the embodiment of price hikes on off-patent essential medicines (Alpern et al., 2019). Many of these drugs are widely available and low-cost in the developing world where tropical parasitic infections are endemic, but have become more expensive in the United States where these infections have relatively low incidence and prevalence. In this article, we describe the pricing and market conditions of two anti-parasitic drugs: albendazole and miltefosine.

Whereas albendazole has become the poster child of price hikes on essential off-patent drugs (Alpern et al., 2016), miltefosine was only recently approved by the FDA and has ongoing regulatory market exclusivity. Although very different drugs with respect to their market and regulatory conditions, both drugs are used to treat neglected tropical diseases that are relatively uncommon in the US: hydatid disease, neurocysticercosis, and soil-transmitted helminth (STH) infections (albendazole) and leishmaniasis (miltefosine).

## Albenza (Albendazole)

In October 2019, a 12-year old girl, who recently arrived in Minnesota from Ecuador, presented to a free clinic with diffuse abdominal pain and fatigue. She was diagnosed with hookworm infection—a soil-transmitted helminth (STH) that affects over 500 million people worldwide (Hotez et al., 2004). Like other STH infections, hookworm has a much higher prevalence in areas of extreme poverty—predominantly in the developing world—where a common mode of transmission is walking barefoot on soil (Hotez et al., 2004; CDC, 2013). Hookworm infection can cause chronic blood loss and may reduce school attendance in children, with subsequent effects on productivity and wage-earning potential in adulthood (Hotez et al., 2004). The clinic is usually able to provide free prescription medicines to the patients it serves, many of whom are uninsured or underinsured. In this case, however, the average wholesale price (AWP) of the first-line treatment—a single dose of 400 mg albendazole—was over $400 (Dynamed Plus, 2020; Micromedex 2.0, 2020). At the time, the lowest price available with a coupon discount on GoodRx was still prohibitive for both the clinic and the patients' family. Instead, she was prescribed a trial of pyrantel pamoate, a less-effective over-the-counter alternative which failed to resolve the infection. Out of options, the free clinic referred the patient to a nearby federally-qualified health center, hoping she would be able to access albendazole or mebendazole through a public program.

The antiparasitic medication, albendazole, has been marketed outside the US since 1982 and was approved by the FDA in 1996. In addition to the treatment of hookworm (*ancylostoma duodenale* and *necator americanus*) it is first-line for the treatment of neurocysticercosis and echinococcosis and is a preferred treatment option for *ascaris lumbricoides* and pinworm (*enterobius vermicularis*)—the most common parasitic infection in the US (CDC, 2013).

In 2010, CorePharma acquired the marketing license for Albenza (albendazole) from GlaxoSmithKline (GSK) and then sold the drug to Amedra Pharmaceuticals, a private equity firm. Amedra then purchased the primary competitor in the US market, mebendazole. Between 2010–2015, the AWP of Albenza increased by 3,299%, from $5.92 per 200 mg tablet in 2010 to $201.27 in 2015 (Alpern et al., 2016). In 2015, Impax Labs (now Amneal Pharmaceuticals Inc.) acquired Amedra. This led to subsequent increases in the price of Albenza, eventually landing on its current average wholesale price of $291.21 per tablet, and bringing the total increase in AWP since 2010 to 4,819% (Micromedex 2.0, 2020). It took until September of 2018 for the first generic manufacturer to begin to market albendazole in the US, and since then five other companies have entered (FDA, 2020). According to a recent FDA report, drug prices decline to ~47% of brand-name drug prices with 2 generic manufacturers, 32% with 3 manufacturers, and 14.4% with 5 manufacturers (Food and Drug Administration, 2019a).

While historical data are limited, between January 2018 and August 2020, the lowest out-of-pocket price of a single 200 mg tablet of albendazole (half of the first-line 400 mg treatment dose for hookworm) after the use of GoodRx coupons decreased from $191 to $49 (GoodRx, 2020; Wayback Machine, 2020). Interestingly, despite the presence of multiple generic manufacturers and the decline of the GoodRx post-coupon price for patients, the average wholesale price (AWP) of generic albendazole remains high. As of August 2020, the mean AWP of a single 200 mg tablet, among the five available generic products, is $248 or 85.2% of the brand-name (Micromedex 2.0, 2020). Although the AWP is not a good measure of the price actually paid for a drug, it can translate to high out-of-pocket costs for patients—particularly the uninsured. One possible explanation for this observation is that insufficient time has passed to realize the effect of competition on price. However, some evidence suggests that a more significant AWP reduction for generic albendazole tablets should have been observed by now (IMS, 2016; Food and Drug Administration, 2019c). Although prices do not appreciably decline after the entry of one generic manufacturer, prices typically decrease rapidly with the entry of subsequent generic manufacturers (Food and Drug Administration, 2019c; Gupya et al., 2019).

The delayed entry of manufacturers for generic albendazole could reflect FDA policy in the last few years to incentivize generic entry in non-competitive markets. Since 2017, the FDA has maintained a list of off-patent off-exclusivity (OPOE) drugs with one manufacturer in the US in order to encourage generic entry for candidate drugs (Food and Drug Administration, 2019a). Under the FDA Reauthorization Act of 2017 (FDARA) Congress also created a competitive generic therapy (CGT) designation for Abbreviated New Drug Applications (ANDAs) in drug markets with only one manufacturer (Food and Drug Administration, 2019b). The CGT designation allows for an expedited and prioritized review process, as well as eligibility for a 180-day period of market exclusivity (Food Drug Administration, 2018). As of March 2019, the FDA had received more than 245 CGT requests and granted over 70% of them (Food and Drug Administration, 2019b).

## Impavido (Miltefosine)

Miltefosine is the only oral drug FDA-approved to treat leishmaniasis, a parasitic disease that can present in a cutaneous, mucocutaneous, or visceral form. Untreated, the visceral form has a high mortality (Sunyoto et al., 2018) and causes 20,000–30,000 deaths annually (WHO, 2019). Stigma and disability due to cutaneous and mucocutaneous lesions can be devastating (Hofstraat and van Brakel, 2016). Miltefosine costs ~$57,600 for a 28-day regimen in the US, resulting in barriers to access due to high out of pocket costs (WHO, 2019). In contrast to albendazole, which has generic manufacturer competition, the sole manufacturer of miltefosine has orphan drug market exclusivity through March 2021.

The effort to bring miltefosine to the US market for the treatment of leishmaniasis began in 2008 when Paladin Labs acquired the rights to the drug from Zentaris for $8.5M. Between 2008 and 2014, Paladin acted as the drugs' sponsor and spent roughly $10M working toward FDA approval (Doshi, 2014). In late 2013, Paladin Labs was acquired by Endo Pharmaceuticals for $1.6B (WHO, 2019). By this point, Paladin's new drug application for miltefosine was nearing approval and Paladin placed a $100M+ price tag on miltefosine, a price Endo was unwilling to pay (Doshi, 2014). Thus, as Endo absorbed Paladin, Knight Therapeutics—led by the CEO of Paladin—was spun off in February 2014 with worldwide rights to miltefosine. Less than a month later, miltefosine was approved by the FDA for the treatment of leishmaniasis, granting Knight a tropical disease priority review voucher (PRV). The tropical disease PRV is a reward meant to incentivize research and development (R&D) for neglected tropical disease (NTD) drugs. Since the conception of the PRV program in 2007, if a sponsor achieves approval for a new chemical entity that constitutes a significant improvement for one of the listed tropical diseases, the sponsor can be granted a PRV (Kesselheim et al., 2015). The voucher is redeemable at the FDA for the priority review (as opposed to standard review) of a different drug or biologic product, and may also be transferred or sold—with market value estimates as high as $350M at the time (2017) (WHO, 2019).

Only 5 months after being granted the PRV, Knight Therapeutics sold the voucher in November, 2014 to Gilead for US $125M in cash, well before the drug was made available in the US market (DNDi, 2014; Garde, 2014; Knight Therapeutics Inc, 2014). The drug did not enter the US market until April 2016, priced at an average wholesale price of US$685.70 per capsule, or roughly US$57,600 for a 28-day regimen (84 capsules).

The case of miltefosine provided some of the earliest evidence that the PRV may not be driving research and development of tropical drugs it had originally intended. Manufacturers are able to bring an existing drug to the US market while avoiding some or all of the research and development costs and receive a tropical disease PRV—which can be sold for a profit (Kesselheim et al., 2015), arguably over-compensating the manufacturer. In addition to the profits enjoyed from the sale of the PRV, companies who commercialize orphan drugs are also granted market exclusivity of up to 7 years, giving them the ability to demand high prices. Global experts have suggested that preconditions on PRVs should stipulate that applicants seek regulatory approval of the drug in endemic countries and demonstrate appropriate access strategies (WHO, 2019).

## Looking Ahead

Some segments of the US antiparasitic drug market have been targeted by a pharmaceutical industry increasingly focused on financialization and short-term returns. This business model is troubling to healthcare providers because it seems that vulnerable patients have been disproportionately affected (Hotez, 2014; Alpern et al., 2016). The examples of albendazole and miltefosine highlight different but equally important ways in which the US drug development mechanism has failed patients.

The neglected tropical disease PRV program has been in effect for over a decade. The story of Miltefosine provides some evidence that the program may be functioning sub-optimally. An analysis of NTD drug development in the 7-year period preceding and succeeding the PRV program's conception demonstrated no association between the NTD PRV program and an increase in innovative, early-stage NTD product development (Jain et al., 2017). This finding stands in contrast to other markets, such as drugs used to treat rare pediatric diseases, where a similar PRV program has demonstrated some effect on early-stage drug development (Hwang et al., 2019). If the PRV program is to persist in the NTD space, requirements specific to the NTD market conditions may be warranted. For example, the FDA could hold manufacturers accountable for certain access benchmarks and consider withholding the voucher until the drug has been become available at what is deemed to be a fair price.

In the case of albendazole, generic entry alone may not be sufficient to lower prices significantly. If similar trends are identified in other drug markets, additional policies may be needed. While this piece was being written, one of the authors was traveling in Ecuador for the holiday and visited a licensed local pharmacy. There, a 400 mg dose of albendazole (FAGOL 400), the first-line treatment for hookworm (Dynamed Plus, 2020), sold for $0.33 USD—less than a pack of gum. In Minnesota, as of August 2020, the lowest out-of-pocket price (after coupons) of the same 400 mg dose is still $98.46 (GoodRx, 2020). It is indeed a paradox that a patient from Ecuador seeking healthcare at a free clinic in Minnesota would be better served receiving this care in Ecuador, where the retail out-of-pocket cost of first-line treatment is <0.5% of what it is here.

## Data Availability Statement

The original contributions presented in the study are included in the article/supplementary material, further inquiries can be directed to the corresponding author.

## Author Contributions

AS: research and preparation of the manuscript. JA: critical review and preparation of the manuscript. Both authors contributed to the article and approved the submitted version.

## Conflict of Interest

The authors declare that the research was conducted in the absence of any commercial or financial relationships that could be construed as a potential conflict of interest. The handling editor declared a shared affiliation with the authors at time of review.
